# Stress level and heart rate variability in police post-occurrence of bank robbery: an experience report

**DOI:** 10.47626/1679-4435-2022-697

**Published:** 2023-02-03

**Authors:** Gabriel Grani, Julio César Bassan, Elisangela Franciele Rezende, Henrique Lubas, Leonardo Farah, Roberta Luksevicius Rica, Danilo Sales Bocalini, Carlos Alexandre Molena Fernandes, Anderson Caetano Paulo

**Affiliations:** 1 Departamento Acadêmico de Educação Física, Universidade Tecnológica Federal do Paraná, Curitiba, PR, Brazil.; 2 Batalhão de Operações Especiais, Polícia Militar do Paraná, Curitiba, PR, Brazil.; 3 Educação Física, Centro Universitário Campos de Andrade, Curitiba, PR, Brazil.; 4 Educação Física, Universidade Estácio de Sá, Vitória, ES, Brazil.; 5 Educação Física, Universidade Federal do Espírito Santo, Vitória, ES, Brazil.; 6 Educação Física, Universidade Estadual do Paraná, Paranavaí, PR, Brazil.

**Keywords:** occupational risks, physical ftness, coping skill, health of military personnel, autonomic nervous system, riscos ocupacionais, aptidão física, estratégia de *coping*, saúde militar, sistema nervoso autônomo

## Abstract

**Introduction:**

The expectation of armed confrontation is among the most stressful elements in police work. Knowledge about perceived stress and cardiovascular markers in police officers comes from simulations. However, to date, information about psychophysiological responses during high-risk occurrences is scarce.

**Objectives:**

To assess stress levels and heart rate variability in policemen before and afer atending a bank robbery.

**Methods:**

Elite police officers (30.4 ± 3.7yrs) filled in a stress questionnaire and had their heart rate variability monitored at the beginning (7:00 am) and at the end (7:00 pm) of a work shif. At about 5:30 pm, these policemen were called to respond to a bank robbery in progress.

**Results:**

No significant changes in sources or symptoms of stress were found between before and afer the incident. However, statistical reductions were found in heart rate range interval (R-R interval [-13.6%]), pNN50 (-40.0%), and low frequency (-28%) and the low frequency/high frequency ratio increased (200%). These results suggest that although no change in the level of perceived stress was found, a significant reduction in heart rate variability may be atributed to a reduction in activation of the parasympathetic system.

**Conclusions:**

The expectation of armed confrontation is among the most stressful occurrences in police work. Research knowledge about perceived stress and cardiovascular markers in police officers comes from simulations. Data on psychophysiological responses post-occurrence of high-risk scenarios are scarce. This research may help law enforcement organizations find means to monitor police officers’ acute stress levels afer any high-risk occurrences.

## Abstract (pt)

**Introdução:**

No trabalho policial, a expectativa do confronto armado está entre as ocorrências mais estressantes. O conhecimento sobre as alterações dos níveis de estresse e marcadores cardiovasculares são oriundas de condições simuladas. Até o momento, a descrição das reações psicofsiológicas após ocorrência de alto risco são escassas.

**Objetivos:**

Verificar o nível de estresse e a variabilidade da frequência cardíaca em policiais após uma ocorrência de roubo a banco.

**Métodos:**

Oito policiais de elite (30,4±3,7 anos) preencheram um questionário de estresse e tiveram sua variabilidade da frequência cardíaca monitoradas no início (7h) e no final (19h) do turno de trabalho. Entretanto, por volta das 17h30, esses policiais atenderam uma ocorrência de roubo a banco.

**Resultados:**

Não foram encontradas alterações significantes entre o início e o final do plantão para as fontes e sintomas de estresse. Entretanto, houve redução significante para o intervalo da variação de frequência cardíaca [intervalo R-R (-13.6%)], pNN50 (-40.0%), baixa frequência (-28%) e aumento para razão baixa frequência/alta frequência (+200%). Dessa forma, nossos resultados sugerem que embora nenhuma mudança no nível de estresse percebido tenha sido encontrada, uma redução importante na variabilidade da frequência cardíaca pode ser atribuída a uma redução na ativação do sistema parassimpático.

**Conclusões:**

A associação de indicadores qualitativos e quantitativos (questionário e variabilidade da frequência cardíaca) contribui para um melhor entendimento de como gerenciar e treinar as habilidades emocionais dos policiais. Esta pesquisa pode ajudar as organizações de aplicação da lei a encontrar propostas para monitorar os níveis de estresse agudo dos policiais após qualquer ocorrência de alto risco.

## INTRODUCTION

There is consensus in studies on occupational stress^1^ that the police is one of the professions that involves the greatest sources of stress, risk of accident, and risk of death.^2^,^3^ Many studies have reported occupational factors of this profession: including burnout syndrome, depressive symptoms, poor quality of life, multi-site musculoskeletal pain, excessive bureaucratic procedures, worry about family members, lack of media and popular support, and traumatic incident experiences.^1^,^4^ Despite the consensus that exists on chronic stress, there is litle knowledge about changes in acute stress in police officers afer high-risk incidents. Furthermore, researchers have highlighted the need to acquire knowledge using quantitative approaches.^5^,^6^

Stress is a relationship between the perceived threat of an emotional, physical, or psychosocial demand and the assessment of the ability to respond to this demand. An acute imbalance in the level of stress can trigger reactions in the organism’s psychophysiological systems that can afect the police officer’s physical and mental balance,^7^ puting at risk the whole team’s physical safety and the success of the operation. This is why it is important to monitor stress levels in order to evaluate the psychophysiological changes that Elite police officers undergo during real operational occurrences.

In terms of psychometric changes, it has been recognized that the response to stress is not an isolated result of the particular moment - the intensity of the response can be associated with previous training factors, previous experiences, and with levels of satisfaction with basic, physiological, social, and afective needs, and achievement.^8^ To assess these, Rushall^9^ developed a psychometric measure to quantify the source and symptoms of stress at a given moment, based on a questionnaire called Daily Analysis of Life Demands in Athletes (DALDA). This questionnaire has already proved useful for evaluating military personnel.^10^

Regarding quantitative changes, it is known that stress levels may be associated with the sympathovagal balance of people in professions such as the police.^6^,^11^ The parasympathetic nervous system is inhibited and the sympathetic nervous system stimulates the adrenal glands to release adrenaline, increasing heart rate (HR), blood pressure, frequency, and respiratory depth, resulting in redistribution of blood flow to the muscles, preparing the body for situations of fight and fight. One method of inference for this sympathovagal balance is to measure HR variability (HRV) in both time and frequency domains.^12^ Strahler & Ziegert^6^ demonstrated a reduction in HRV afer a reality-based school shooting simulation. However, almost nothing is known about the cardiovascular changes of police officers in real emergency situations.

One of the operational demands that cause acute stress to a police officer is the expectation of confrontation with armed criminals. In Brazil, the police officers responsible for atending this type of occurrence belong to an elite group named the Special Operations Batalion (BOPE),^13^ who undergo arduous training in emotional control. It is important to understand the effects on these policemen’s psychophysiological behavior of atending a high risk operational incident.

The aim of the present study was to compare stress levels and HRV before and afer atending a bank robbery. We hypothesized that the BOPE police would not exhibit a significant increase in stress levels and that there would be a significant reduction in HRV at the end of the shif. This combination indicates an optimal state of alertness for performing their job functions.

## METHODS

### STUDY POPULATION

This is a cross-sectional, descriptive study. The sample was recruited by invitation and convenience on a day chosen for the BOPE study. The experimental procedures were approved by the local police Commander and by the local Research Ethics Commitee (Certificate of Presentation for Ethical Appraisal n. 2.133.438/2017). All participants signed Free and Informed Consent Forms, complying with the norms of the National Health Council (Resolution number 466/2012).

The outcome variables of the study were comparison of DALDA psychological questionnaire scores and changes in the subjects’ HRV from the beginning to the end of the work shif.

### POLICE STRESSOR MEASURE

The DALDA is made up of two parts. Its purpose is to identify changes in sources and symptoms of stress at a given moment.^9^ The first part is composed of nine questions that measure sources of stress. The second part contains 25 questions that identify symptoms of stress. Conceptually, the source of stress would be the emergence of a threat and coping with the stressful condition, such as pursuit of a suspect. The stress symptoms would be related to changes in emotions (i.e. pleasure, fear, indiference, fatigue, aggressiveness, volition) when faced with a source of stress. An arbitrary score was assigned to each of the three emotional response options (a = worse than normal; b = normal; c = beter than normal) with 3 points for a, (negative response); 2 points for b; and 1 point for c (positive response). For purposes of analysis, the overall sum of the responses to each part of the scale was used. The internal consistency of the stress questionnaire has been verified previously with Cronbach’s α (DALDA, Part A = 0.73, Part B = 0.72)^14^ and the Portuguese version has been validated.^15^ However the DALDA was modified for our study, since we changed two questions. In question 5, part A, “Sport training” was changed to “Tactical training” and in question 15, part B, “Between sessions recovery” was changed to “Between work shif recovery”.

### HEART RATE VARIABILITY

Changes in the HR range interval (R-R) were recorded using a Polar T 31 coded belt. This belt transmits pulses to a WCS Pulse receiver.^16^ For capture, the receiver was calibrated to detect radiofrequency pulses in the range of 5 KHz (+/- 100 Hz). Each receiver is equipped with a universal serial bus (USB) cable that transmits the data received to a computer containing the WCS Pulse sofware and archives the R-R intervals in millisecond format in txt format. This procedure followed all of the recommendations of the European Society of Cardiology (ESC) and North American Society of Pacing and Electrophysiology (NASPE).^12^

HRV Kubios Sofware was used to determine HRV temporal and spectral variables. In the time domain, the mean normal interval (mean R-R) was calculated and the root mean square of successive diferences between adjacent normal R-R intervals (RMSSD) and the percentage of adjacent R-R intervals with a duration diference greater than 50ms (pNN50) were derived. In turn, the spectral variables analyzed were LF (low frequency = 0.04 - 0.15 Hz), which is associated with the sympathetic system; HF (high frequency = 0.15 -0.40 Hz), which is associated with the parasympathetic system; and the LF/HF ratio (sympathovagal balance).

### EXPERIMENTAL PROCEDURES

Each policeman was invited to participate early in his morning shif. Afer signing a Free and Informed Consent Form, he was sent to a room, which is in a quiet place in the headquarters of the General Command of the city of Curitiba - PR, Brazil. Here, the policeman remained seated to respond to the Modified DALDA; then he had a coded model T 31 Polar belt fted to his chest. The belt transmiter was moistened to facilitate capture of the R-R intervals. The policeman then assumed the supine position on a mat while his HR was monitored for a period of 5 minutes. During this period the participants were instructed not to sleep and to avoid voluntary changes in breathing rhythm (yawning or deeply inhaling). This data collection took place between 7:00 AM and 7:30 AM. Afer the initial data collection, the BOPE police officers returned to their normal work routines.

At around 5:30 p.m. that day, the Military Police Operations Center reported that a bank robbery was in progress in the city of Colombo, PR. This city is located 15 km from the headquarters of the General Command and all the military police who volunteered to participate in this study were assigned to deal with this incident. For security reasons, the researchers were not given access to details of the incident. It was reported to us, however, that there was no armed confrontation and the robbers had already fed from the crime scene.

Afer atending the incident, the police officers returned to the General Command headquarters to finish their work shifs. As they arrived, they were taken to the same room, where they were again subjected to the same procedures as in the morning, that is to respond to the Modified DALDA questionnaire and to have their HRV measured. This second data collection session took place between 7:30 and 8:00 p.m.

### STATISTICAL ANALYSIS

Descriptive statistical analyses (mean, standard deviation [SD], percentage) were conducted on the data from the beginning and end of the work shif. The normality and homogeneity of the data were examined with the Shapiro Wilk test. The Wilcoxon test was used to identify changes in stress sources (part 1) and symptoms (part 2), as recorded in the DALDA responses; and the paired *t* test was used to identify changes in HR and HRV parameters. The effect size was calculated with the following formula: Cohen’s d = Post-Occurrence mean – Pre-Occurrence mean/SD_pooled_, where $SD_{\text{pooled}}=\sqrt{}\left[SD_{\text{post-occurrence}}{}^{2}+SD_{\text{pre-occurrence}}{}^{2}/2\right]$ .^17^ The resulting effect size was classified as Small (0.00 ↔ 0.50), Moderate (0.51 ↔ 1.0), or Large (> 1.0). A 5% level of statistical significance was adopted in all analyses (p < 0.05). Statistical calculations were performed using the Statistical Package for Social Sciences (SPSS), version 22.0.

## RESULTS

Eight military police officers participated in the study, with a mean age of 30.4 ± 3.7 years; body mass of 91.5 ± 13.5 kg; mean height of 1.80 ± 0.06 cm; and a mean service time of 7.4 ± 4 years as a police officer and 4.0 ± 3.8 of these years in the BOPE division. All participants atended the bank robbery incident.

On the sources and symptoms of stress, the Wilcoxon test revealed that there was no significant change at any time when they were on call (Table 1).

**Table 1:** Sources and symptoms of stress in elite military police officers before and after the occurrence of a bank robbery

	Pre-occurrence (Σ)	Post-occurrence (Σ)	Z	p-value	Cohen’s d
Stress source	16.37 ± 1.06	16.50 ± 1.76	0.14	0.89	0.09 (small)
Stress symptoms	48.37 ± 2.26	48.12 ± 4.91	0.21	0.83	-0.07 (small)

The frequency of ‘beter than normal’, ‘normal’, and ‘worse than normal’ responses was also similar between the beginning and end of the shif (Table 2).

**Table 2:** Frequency of ‘better than normal’, ‘normal’, and ‘worse than normal’ responses to Daily Analysis of Life Demands in Athletes (DALDA)-modified

	Worse than normal (%)		Normal (%)		Better than normal (%)	
	Pre	Post	Pre	Post	Pre	Post
Part A						
1. Diet	3 7.5	3 7.5	62.5	62.5	0.0	0.0
2. Home-life	0.0	0.0	100.0	100.0	0.0	0.0
3. School/college/work	0.0	0.0	100.0	100.0	0.0	0.0
4. Friends	0.0	0.0	100.0	8 7.5	0.0	25.0
5. Tactical training*	3 7.5	25.0	62.5	75.0	0.0	0.0
6. Climate	0.0	12.5	100.0	8 7.5	0.0	0.0
7. Sleep	75.0	50.0	25.0	50.0	0.0	0.0
8. Recreation	12.5	12.5	8 7.5	8 7.5	0.0	0.0
9. Health	0.0	0.0	100.0	100.0	0.0	0.0
Stress sources	18.1	18.1	81.9	80.6	0.0	1.4
Part B						
1. Muscle pains	12.5	0.0	75.0	100.0	12.5	0.0
2. Techniques	0.0	12.5	100.0	8 7.5	0.0	0.0
3. Tiredness	37.5	12.5	62.5	8 7.5	0.0	0.0
4. Need for a rest	37.5	50.0	62.5	3 7.5	0.0	12.5
5. Supplementary work	37.5	0.0	62.5	100.0	0.0	0.0
6. Boredom	12.5	0.0	75.0	8 7.5	12.5	12.5
7. Recovery time	12.5	12.5	87.5	8 7.5	0.0	0.0
8. Irritability	0.0	0.0	87.5	8 7.5	12.5	12.5
9. Weight	37.5	37.5	62.5	62.5	0.0	0.0
10. Throat	0.0	0.0	8 7.5	100.0	12.5	0.0
11. Internal	0.0	0.0	100.0	100.0	0.0	0.0
12. Unexplained aches	0.0	0.0	62.5	75.0	37.5	25.0
13. Technique strength	12.5	12.5	8 7.5	8 7.5	0.0	0.0
14. Enough sleep	75.0	75.0	12.5	25.0	12.5	0.0
15. Between work shift recovery*	0.0	0.0	100.0	100.0	0.0	0.0
16. General weakness	0.0	12.5	75.0	75.0	25.0	12.5
17. Interest	12.5	0.0	87.5	100.0	0.0	0.0
18. Arguments	37.5	0.0	62.5	87.5	0.0	12.5
19. Skin rashes	12.5	0.0	62.5	100.0	25.0	0.0
20. Congestion	12.5	0.0	87.5	100.0	0.0	0.0
21. Training effort	12.5	25.0	87.5	75.0	0.0	0.0
22. Temper	0.0	0.0	87.5	100.0	12.5	0.0
23. Swellings	12.5	0.0	62.5	100.0	25.0	0.0
24. Likeability	0.0	0.0	100.0	100.0	0.0	0.0
25. Running nose	12.5	0.0	75.0	100.0	12.5	100.0
Stress symptoms	15.5	10.0	76.5	86.5	8.0	3.5

* Questions modified for police officers.

Regarding HR, the *t* test revealed a statistically significant diference in HR and some HRV indicators (Table 3). HR increased 14.4%, mean R-R decreased -13.6%, pNN50 decreased -40.0%, LF (Hz) decreased -28%, and LF/HF ratio increased by almost 200%. The other changes were not statistically significant.

**Table 3:** HRV of elite police officers before and after the occurrence of a bank robbery

Parameters	Pre-occurrence	Post-occurrence	95%CI		T value	p-value	Cohen’s d
Heart rate (bpm)	70.45 ± 9.81	80.62 ± 8.74*	-19.84.	-0.50	-2.49	0.04	-0.88 (large)
R-R median (ms)	879.62 ± 137.88	759.35 ± 87.20*	-5.59	246.14	2.26	0.05	0.80 (large)
RMSSD (ms)	62.54 ± 32.15	50.56 ± 53.57	-37.56	61.52	0.57	0.59	0.20 (small)
pNN50 (%)	30.50 ± 24.69	12.35 ± 11.08*	-4.57	40.87	1.89	0.04	0.67 (medium)
LF (Hz)	0.11 ± 0.03	0.08 ± 0.03*	0.00	0.05	2.67	0.32	0.95 (large)
HF (Hz)	0.24 ± 0.06	0.24 ± 0.10	-0.10	0.10	-0.05	0.96	-0.02 (small)
LF (n.u.)	51.85 ± 16.5	66.9 ± 18.90	-32.14	1.99	-2.09	0.07	-0.74 (medium)
HF (n.u.)	4 7.9 3 ± 16.38	39.11 ± 22.48	-16.32	33.96	0.83	0.43	0.29 (small)
LF/HF	1.35 ± 0.93	2.68 ± 1.40*	-2. 34	-0.33	-3.13	0.02	-1.11 (large)

* Different pre-occurrence p < 0.05.

95%CI = 95% confidence interval; HF = high frequency; HR = heart rate; HRV = heart rate variability; LF = low frequency; n.u. = normalized units; RMSSD = root mean square of successive diferences; R-R = HR range interval.

## DISCUSSION

The present study verified the impact of being called to the scene of a bank robbery on the acute responses of stress levels and indicators of autonomic regulation in elite policemen. The main results revealed that there was no change in the stress level, but there was a reduction in HRV. These findings corroborate our initial hypotheses. It is not known to the authors if there is another similar study in the literature.

Several studies have shown that stress levels in police officers are related to conflicts with their spouses,^18^ poor communication with police supervisors and peers,^19^ lack of social support and demographic characteristics,^20^ and organizational and operational routines.^21^ There is a knowledge gap about acute stress changes afer a high-risk incident.

It can be stated that a confrontation with heavily armed robbers is among the most dangerous of operational demands.^6^ High-speed motorized pursuit,^3^ negotiating with criminals,^22^ hostage rescue,^23^ and possible shootings^24^ are prevalent in bank robberies. In spite of this high emotional demand, however, the policemen in our sample did not exhibit changes in the level of perceived stress. Likely, this stress control was related to the characteristics of the police in our sample or the specifics of the incident in question.

Regarding the police officers’ characteristics, we observed that more than 75% of the sample had already endorsed sources and symptoms of stress as ‘worse than normal’ at the beginning of the shif (Table 2). Additionally, poor quality of sleep was mentioned by all of the police officers who reported a high level of stress at the start of the shif. Moreover, the modified DALDA questionnaire does not measure chronic or acute stressors separately, rather a mixture of the two, which may explain the high level of stress at the start of the shif. This is in line with other studies.^8^,^25^,^26^ However, the BOPE police’s stress levels did not change post-occurrence, which suggests adequate coping strategies.^7^,^25^ Coping means investing a conscious effort to solve problems, trying to master, minimize, or tolerate conflicts and stress. Studies have revealed that experienced police officers and enlisted men present beter coping strategies to deal with stress levels than female police officers and novices.^25^,^27^ A recent study also showed that cognitive-emotional intervention and decision-making profiles diferentiated between Special Weapons and Tactics (SWAT) officers and negotiator squad operators for the same incident.^22^ Like SWAT officers, BOPE police officers are trained in precision shooting, dynamic building entry, and endurance for callout lasting over 12 hours. On the other hand, the negotiator squad is trained to convince a person to surrender peacefully. Our sample was composed of male police officers, with 7 years of experience, and all members of BOPE units. BOPE police officers are called upon to respond to the demands of routine police patrols and must also be prepared for armed confrontations, violent situations, civil unrest or prison, search and seizure of drugs or fugitives from justice, and to act against terrorism.^13^ So, even though the BOPE police officers might have experienced some stress symptoms during the robbery, afer their return to the headquarters they may have felt relieved and, therefore, not reported any symptoms.

Regarding the specifics of the incident, the Paraná State Public Security Secretariat reports an average of 0.24 robberies per bank per municipality in a 1-year period. Atendance at this type of incident positively interferes in police officers’ stress levels. A group of 617 Italian police officers and 47 Canadian police officers answered two police stress perception questionnaires.^21^,^25^ The first questionnaire was related to administrative routines such as dealing with supervisors, performing bureaucratic duties, waiting in courts or police stations, and conducting theoretical courses on new laws and procedures. The second questionnaire was related to operational routines such as how to approach a suspect, atendance at incidents, and dealing with risks. The results of the two studies showed that in addition to administrative activities occurring at a higher rate than operational activities, Italian and Canadian police perceive administrative activity as more stressful.^21^,^25^ Thus, being called to an incident such as a bank robbery extricates BOPE officers from the administrative routine. Informal reports suggested that the policemen in the sample were euphoric at the end of the shif.

In turn, HRV may be a quantitative indicator of both emotional and physical stress levels in military police officers. Successful short-term cardiovascular regulation allows immediate adjustments of HR and blood pressure in situations of acute modification of emotional or physiological demands.^6^,^11^ Robust activation of the sympathetic nervous system associated with an abrupt reduction of the parasympathetic nervous system can result in negative cardiovascular outcomes such as arrhythmias, dizziness, tremors and, in extreme cases, sudden death.^28^ In fact, there are reports that 45% of deaths of police officers and firefighters in service are caused by cardiovascular problems.^29^ One of the explanations for this outcome would be associated with a high emotional and physical demand with poor cardiovascular regulation.

The cardiovascular indicators measured at the beginning of the shift suggest normal functioning for our sample (Table 3). Even an average value of 62 ms for the RMSSD is indicative of a good level of fitness. The RMSSD measurements are sensitive under conditions of improved vagal outlet flow, particularly in the supine position.^30^ Furthermore, Strahler & Ziegert^6^ showed that RMSSD returned to baseline within 25 min after a simulated school shooting. This result was similar to our study because there was also no difference between pre-occurrence and post-occurrence RMSSD. However, at the end of the day, there was a reduction in the mean R-R and the pNN50, indicating tachycardia and an attenuation of vagal modulation, respectively. LF (Hz) and an increase in the LF/HF ratio indicate that HRV reduction due to a reduction in the activation of the parasympathetic system did not occur due to a significant increase in the sympathetic system. This behavior suggests normal physical fatigue at the end of the shift, rather than emotional stress as a result of the bank robbery. Reduced HRV at the end of a shift has also been demonstrated in firefighters.^31^

There is a previous study that monitored psychophysiological responses to stress levels and heart rate in other professionals working in high-risk situations.^32^ However, field studies with real-life stressors are scarce in the police literature and our study captured (acute) stress responses to a real-life police incident with a difficult-to-access, highly specialized sample. Investigation of police officers’ psychophysiological stress responses to real-life critical incidents is extremely important to understanding the short-term and long-term effects of stress on work performance and health. Field studies in general, and in high-risk profession in particular, are naturally accompanied by logistic limitations in stress measurement and we therefore classify our manuscript as an experience report.

Despite its limitations, listed below, our study serves as a starting point for future research. First, our sample comprised just eight elite military police officers, so our results cannot be extrapolated to another type of police officer, suggesting that future studies might, if possible, be carried out with a larger sample. Second, in this bank robbery incident, the robbers fed the crime scene, so there was no direct confrontation with the robbers, who were the primary stress agents. Tird, we did not measure the coping strategies adopted by the police officers. Fourth, two questions on the DALDA were modified for police officers. Thus, future research may: 1) analyze larger samples and include police officers with different qualifications; 2) verify occurrences where there is direct confrontation with agents of stress, with or without success in the operation; 3) evaluate and re-evaluate the coping strategies employed by the same police officer in different situations; and 4) develop and apply questionnaires more specific to elite police officers.

## CONCLUSIONS

The elite BOPE squad police officers did not suffer a significant increase in stress levels and exhibited a significant reduction in HRV at the end of the shift. This combination may indicate an optimal state of alertness for performing their job functions. This research may help law enforcement organizations find ways to monitor whether their officers’ acute stress levels are adequate to deal with high-risk incidents. In addition, the combination of qualitative and quantitative indicators (questionnaire and HRV) contributes to a better understanding of how to manage and train the emotional skills of military police officers.
